# Molecular cloning and expression analysis of tyrosinases (*tyr*) in four shell-color strains of Manila clam *Ruditapes philippinarum*

**DOI:** 10.7717/peerj.8641

**Published:** 2020-02-17

**Authors:** Kunyin Jiang, Liwen Jiang, Hongtao Nie, Zhongming Huo, Xiwu Yan

**Affiliations:** Engineering and Technology Research Center of Shellfish Breeding in Liaoning Province, School of Fisheries and Life Science, Dalian Ocean University, Dalian, China

**Keywords:** *Ruditapes philippinarum*, Shell-color strains, Tyrosinase, Early development stages, Gene expression, RNAi

## Abstract

The Manila clam (*Ruditapes philippinarum*) is an economically important molluscan bivalve with variation in pigmentation frequently observed in the shell. In nature, tyrosinase is widely distributed in invertebrates and vertebrates, and plays a crucial role in a variety of physiological activities. In this study, a tyrosinase gene (*tyr* 9) was cloned and the expression level of *tyr* genes (*tyr* 6, *tyr* 9, *tyr* 10, and *tyr* 11) were investigated in different shell colors. Quantitative real-time PCR showed that *tyr* genes were significantly expressed in the mantle, a shell formation and pigmentation-related tissue. Moreover, the expression pattern of the *tyr* genes in the mantle of different shell-color strains was different, suggesting that tyrosinases might be involved in different shell-color formation. In addition, the expression profile of *tyr* 6, *tyr* 9, *tyr* 10, and *tyr* 11 genes were detected at different early developmental stages and the expression level varied with embryonic and larval growth. RNA interference (RNAi) results showed that the expression level of *tyr* 9 in the RNAi group was significantly down-regulated compared to control and negative control groups, indicating that *Rptyr* 9 might participate in shell-color formation. Our results indicated that *tyr* genes were likely to play vital roles in the formation of shell and shell-color in *R. philippinarum*.

## Introduction

Mollusks have conspicuous colors and color patterns that attract growing interest from many different perspectives, such as research on shell formation and pigmentation (Meinhardt & Klingler, 1987; Jackson et al., 2010), genetic breeding (Wada & Komaru, 1996), and biomaterial study on pearl formation (Liu et al., 2012). Over the past decades, shell color is widely used as an important trait for selective breeding in many bivalve species, including *Patinopecten yessoensis* (Sun et al., 2015; Liu et al., 2013), *Crassostrea gigas* (Feng et al., 2015), *Hyriopsis cumingii* (Chen et al., 2016), and *Meretrix* (Jing, 2015).

Much of the pigment-based coloration in invertebrates results from the production of the melanin, ommochrome, pteridine, papiliochrome, and heme synthesis pathways (Takeuchi et al., 2005). Of these, melanin is one of the most widespread pigments in nature and consists of two classes: eumelanins and pheomelanins (True et al., 1999). The enzyme tyrosinase is essential for the production of various melanins in invertebrates (Wittkopp, Carroll & Kopp, 2003). In mollusks, tyrosinases are a key compound in shell pigments (Comfort, 2010) and are involved in the regulation of the melanin biosynthesis pathway (Luna-Acosta et al., 2011). In the biosynthesis pathway of melanin, tyrosinase catalyzes three different reactions: (1) the hydroxylation of tyrosine to L-DOPA; (2) the oxidation of L-DOPA to L-dopaquinone; and (3) the oxidation of 5,6-dihydroxyindole to indole-quinone (Sanchez-Ferrer, 1995). Tyrosinases belong to the type-3 copper protein family (Cicero et al., 1982; Johansson & Soderhall, 1996) and possess two conserved copper-binding domains, known as Cu (A) and Cu (B), both of which are coordinated by three conserved histidines (Decker & Tuczek, 2000; Decker et al., 2007).

In recent years, the genetic bases and molecular mechanisms of shell and shell-color formation are receiving increasing attention (Yue et al., 2015; Rihao et al., 2014). In *Crassostrea angulata*, *Ca-tyrA1* mRNA first occurs at the gastrula stage, persists until the early D-veliger stage and mainly distributes in the mantle of adults (Yang et al., 2017). *Cgi-tyr1* transcripts were first detected in the saddle-shaped shell field in trochophores and were not detected after the D-veliger stage in *Crassostrea gigas* (Huan et al., 2013). It has been reported that the pathways of tyrosinase metabolism and melanogenesis were detected in the mantle transcriptome of *Patinopecten yessoensis*, which indicates that tyrosinase might play a fundamental role in shell pigmentation (Sun et al., 2015). Studies on four shell-color variants of *Crassostrea gigas* have shown that a tyrosinase transcript (CGI_10008737) represented a higher expression level in the golden shell-color variant than in the three other shell-color variants (white, black, and partially pigmented) (Feng et al., 2015). In *H. cumingii*, the activity of tyrosinase in the mantle of a purple strain was significantly higher than in a white strain (Chen et al., 2016). In addition, the expression level of *tyr* genes in a black strain of *Meretrix meretrix* was significantly higher than that in three other strains (white, pink, and red), indicating *tyr* genes were involved in the black appearance of the shell color (Jing, 2015).

*Ruditapes philippinarum*, is an important shellfish with significant economic value, and widely distributed along the coasts of China, Japan, and Korea (Zhang & Yan, 2010). In natural habitats, *R. philippinarum* displays a different shell color, including white, orange, and zebra striated patterns (Nie et al., 2017a). Since 2005, the shell-color strains of the Manila clam were selected for several generations, with the aim of faster growth, stronger resistance, and a high survival rate, and the hybridization of the white and zebra strains (white-zebra strain) has been established (Zhao et al., 2012). In our previous study, 21 tyrosinase genes were found in the *R. philippinarum* genome and six of them were differentially expressed in strains with different colored shells (Yan et al., 2019). However, very few mechanistic studies have been carried out on the shell and shell-color formation of the Manila clam. In this study, a *tyrosinase* (*Rptyr*9) was cloned from *R. philippinarum*, and the relationship between *tyr* genes and various shell colors was investigated by quantitative real-time PCR (qRT-PCR) and RNA interference (RNAi). This study provides new insights on the expression pattern of *tyr* genes in strains of *R. philippinarum* with different colored shells, and the molecular basis of shell-color formation and pigmentation in *R. philippinarum*.

## Materials and Methods

### Experimental Manila clams

Four adult shell-color strains (three dark shell-color strains: orange, zebra and white-zebra clams; and one light shell-color strain: white clam) and a wild population of *R. philippinarum* collected from Zhuanghe and Dalian, China, respectively, were used in the experiment. The color strains of clams were selected by our team (Zhang & Yan, 2010) and wild clam were obtained from commercial sources and harvested by clam collector. Manila clam is not an endangered or protected species, so no specific permits were required for the study (Nie et al., 2017b). The clams had an average shell length of 26.1 ± 2.1 mm and an average weight of 5.85 ± 0.75 g. All the adult Manila clams were acclimatized in aerated seawater (30 ppt) at 20 ± 1 °C and pH 8.1 ± 0.1 for 7 days before the experiment. Clams were fed with Spirulina powder once a day for 1 week and water was exchanged fully once per day to discharge waste products.

For the analysis of *tyr* expression pattern in different shell color strains, the mantle of three adult clams for each shell-color strain were randomly sampled. Different tissues, including mantle, gonad, gill, labial palp, siphon, hepatopancreas, and adductor muscle, were collected from each strain to investigate the tissue-specific expression of *tyr* genes.

### Embryos and larvae collection

The larvae of Manila clams were obtained from the offspring of the wild population, collected from Zhangzi Island (Dalian, Liaoning Province, China). Spawning, fertilization, and embryo collections were performed in controlled lab conditions. The density of fertilized eggs was maintained at 30 eggs mL^−1^ during the incubation period. About 30 h after fertilization, D-shaped larvae were placed into 20 L tanks, at a density of 5–8 individual mL^−1^. Larvae were fed 5,000–20,000 and 40,000–60,000 cells ml^−1^ day^−1^ of *Isochrysis galbana* on days 1–3 and from day 4 to the juvenile stage, respectively. Embryos and larvae at different developmental stages, including egg, fertilized egg, gastrula, trochophore, D-shaped larvae, pediveliger, and juvenile stages, were sampled (Table 1). Collected tissues, embryos, and larvae were immediately frozen in liquid nitrogen and stored at −80 °C until RNA extraction.

**Table 1:** Developmental stages used in the present study.

Developmental stages	Sampling time (after fertilization)
Egg	0 min
Fertilized egg	3 min
Gastrula	6 h
Trochophore	16 h
D-shaped larvae	21 h
Umbo larvae	3 days
Pediveliger	13 days
Juvenile	43 days

### RNA extraction and cDNA synthesis

Total RNA was extracted using TRIzol Reagent (TRIzol^®^ Plus RNA Purification Kit; Invitrogen, Carlsbad, CA, USA), according to the manufacturer’s protocol. The integrity and purity of RNA were determined by electrophoresis on a 1% agarose gel and a Nanodrop ND-2000 spectrophotometer (Thermo Electron Corp., Waltham, MA, USA), respectively. Total RNA was reverse-transcribed to cDNA with a PrimeScript RT reagent Kit (TaKaRa, Tokyo, Japan) and stored at −20 °C before analysis.

### Cloning of the full-length *tyr*9 cDNA

The 5′-untranslated region (UTR) and 3′-UTR of the *tyr*9 gene were obtained by rapid amplification of cDNA ends (RACE) with a SMARTer™ RACE cDNA Amplification Kit (Clontech, Mountain View, CA, USA). The gene-specific primers designed by Primer Premier 5.0 are shown in Table 2. Nest-PCR (Li et al., 2009) and touchdown PCR (Korbie & Mattick, 2008) were used to improve the amplification specificity. The first round of the PCR thermal cycle profile was as follows: five cycles at 94 °C for 30 s, 72 °C for 3 min, and five cycles at 94 for 30 s, 70 °C for 30 s, and 72 °C for 3 min followed by a final five cycles at 94 °C for 30 s, 68 for 30 s, and 72 °C for 3 min. The products were then diluted 50 fold as the template for the second round of PCR. The second round of PCR reaction conditions were 25 cycles at 94 °C for 30 s, 68 °C for 30 s, and 72 °C for 3 min. The PCR products were purified with an agarose gel DNA extraction kit (centrifugal columnar) and cloned into a pMD18-T Simple Vector (TaKaRa, Tokyo, Japan) and then transformed into competent cells of *Escherichia coli* Top10 cells (Tiangen Biotech. Co. Ltd., Beijing, China). Positive colonies containing insert fragments of the expected size were screened by colony PCR. Eight positive colonies were sequenced.

**Table 2:** Primer sequence used in this study.

Primers name	Sequences (5′-3′)	
	Forward primer	Reverse primer
Quantitative real-time PCR primers		
*β-actin*	CTCCCTTGAGAAGAGCTACGA	TAATGACAAGTGGTTTACGGG
*tyr*6	ACCCAGATGAGCGTGGTAGAGG	TTAGTGTTTGGATACGGTGTTG
*tyr*9	ACTGGGATAATACGATAGAAG	GTGCGTTAGGATTAGTTATGT
*tyr*10	ACAGACCAATCACGCAGTTTC	TAGTCTTGCCAAAGCGTCATA
*tyr*11	ATGCGTCAAATGTCTAAATGC	TCTGCGTTTGTGAACTGTGGG
RACE primers		
*tyr* 5′GPS-out	TGGTCCACCATGAGCCGACAGTGCGGTG	
*tyr* 5′ GPS-in	CGCACGCGCAGTTCCCCTTCTGGTGGTA	
*tyr* 3′GPS-out	AACTAATCCTAACGCACCTGATGCCACC	
*tyr* 3′GPS-in	GATGCGACTGGAGTAGACAACGCTGTGT	
Longup	CTAATACGACTCACTATAGGGCAAGCAGT GGTATCAACGCAGAGT	
Shortup	CTAATACGACTCACTATAGGGC	
RNAi primers		
*tyr*F1i	GATCACTAATACGACTCACTATAGGGAATACGATAGAAGAAGGTT	
*tyr*R1	TGTTCTAAAGTCTTCCCAA	
*tyr*F1	AATACGATAGAAGAAGGTT	
*tyr*R1i	GATCACTAATACGACTCACTATAGGGTGTTCTAAAGTCTTCCCAA	

### Sequence and phylogenetic analyses

The cDNA and amino acid sequences of *tyr* were analyzed with the BLAST algorithm at the NCBI website (http://www.ncbi.nlm.nih.gov/blast). The deduced amino acid sequence was analyzed with a Simple Modular Architecture Research Tool (SMART, http://smart.embl-heidelberg.de). Domain searches and annotations were conducted with SMART (Schultz et al., 1998). The amino acid sequence of *tyr*9 that was identified in *R. philippinarum* was compared in a multiple-sequence alignment using DNAMAN (Wang, 2017). Protein sequences were used in the phylogenetic analysis and were aligned by ClustalW with the software package MEGA10 and bootstrapping (*n* = 1,000) (Kumar et al., 2018).

### *Tyr* mRNA expression analysis in different early developmental stages and different tissues of four shell-color strains

Primers used in the study (Table 2) were designed by Primer Premier 5.0 and were synthesized by the Sagon Company (Shanghai, China). Synthesized cDNA template was diluted 10-folds for qRT-PCR. qRT-PCR was carried out on a Roche LightCycler 480 (Roche, IN, USA) using the SYBR ExScript qRT Kit (TaKaRa, Tokyo, Japan), and performed in a total volume of 20 μL, including 10 μL of SYBR^®^ Primix Ex Taq II, 0.8 μL of primer F and primer R, 2 μL of cDNA and 6.4 μL of H_2_O. β*-actin* was performed as an internal control (Nie et al., 2017a). Reactions were performed in 94 °C for 5 min, and 40 cycles of 94 °C for 30 s, 60 °C for 30 s, and 72 °C for 30 s. The purity of amplification products was evaluated by dissociation curve analysis. The 2^−ΔΔCT^ method (Livak & Schmittgen, 2001) was used to analyze the relative expression level of *tyr* genes. The data were the mean ± standard error from at least three independent experiments performed in duplicate. The data were subjected to one-way analysis of variance followed by multiple-range testing in the SPSS 20.0 program. *P* < 0.05 was considered statistically significant.

### The dsRNA synthesis and RNAi of *Rptyr*9

RNA interference primers were designed close to the 5′ end of the *tyr*9 gene sequence of *R. philippinarum*. T7 promoter sequence primer was added to RNAi primers (Table 2). The dsRNA synthesis was performed using an in vitro Transcription T7 Kit for siRNA Synthesis (TaKaRa, Tokyo, Japan) according to the manufacturer’s protocol. The region encompassing positions 536 to 910 of the *tyr*9 cDNA was amplified from the total extracted mRNA, the thermal cycling protocol was 30 cycles at 4 °C for 3 min, 94 °C for 30 s, 68 °C for 30 s; 72 °C for 5 min, 75 °C for 5 min. The PCR amplification products were used as a template for in vitro transcription (Table 3) to synthesize dsRNA. Finally, the dsRNA was analyzed by 1% agarose gel electrophoresis and the quality and quantity were assessed by using a Nanodrop ND-2000 spectrophotometer (Thermo Scientific, Madison, NY, USA).

**Table 3:** In vitro transcription reaction system.

Component	Volume
10 × Transcription buffer	2 μL
ATP solution	2 μL
GTP solution	2 μL
CTP solution	2 μL
UTP solution	2 μL
RNase inhibitor	0.5 μL
T7 RNA polymerase	2 μL
RNase free dH2O	X μL
linear template DNA	20 ng–1 μg
Total	20 μL

In the RNAi experiment, 90 adult wild clams were used and divided into three groups. In the first group, 30 clams were injected into the sinusoid with approximately 100 μL of dsRNA (50 μg mL^−1^) and used as the RNAi group. The control group of 30 clams received an injection of 100 μL phosphate-buffered saline (272 mmol L^−1^ NaCl, 5.2 mmol L^−1^ KCl, 16 mmol L^−1^ Na_2_HPO_4_, 4 mmol L^−1^ KH_2_PO_4_, pH 7.4). The remaining 30 clams were untreated and used as a negative control group. The clams were returned to water tanks after treatment, and three individuals were randomly sampled at 0, 24, 48, 72, 96, and 120 h post-injection from each group. The mantle was collected and then immediately frozen in liquid nitrogen and stored at −80 °C for subsequent RNA extraction.

## Results

### The cDNA cloning and sequence analysis of *Rptyr*9

**Figure 1:**
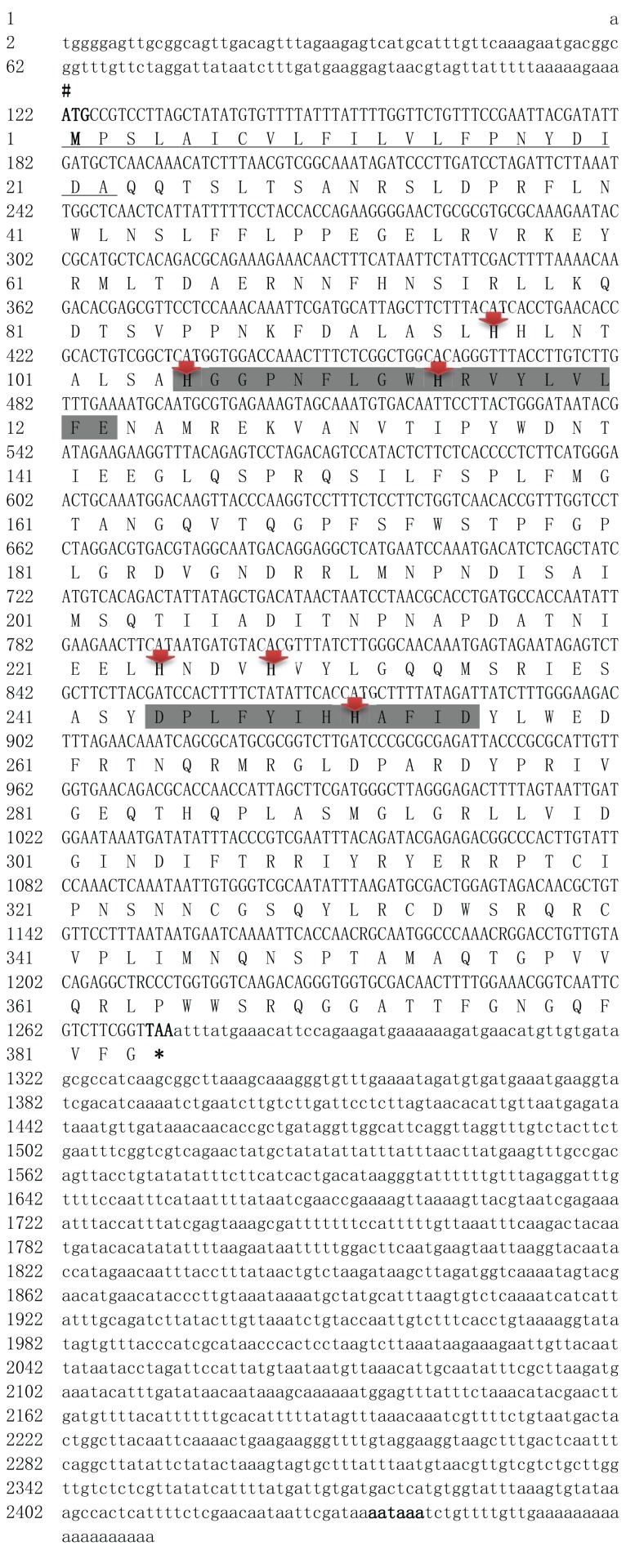
Nucleotide and deduced amino acid sequences of *R. philippinarum tyr* 9. The # and * under the amino acid sequence indicated the promoter sequence (ATG) and translation termination codon (TAA).

The cDNA sequence of the *tyr*9 gene of *R. philippinarum* (designated as *Rptyr*9) was obtained using the RACE method and deposited in GenBank (accession number: MH392190). The full-length cDNA of *Rptyr*9 was 2,452 bp (Fig. 1). As shown in Fig. 1, there is a start codon (ATG) at the 5′ end of the cDNA and a stop codon (TAA) at the 3′ end. *Rptyr*9 contains a 121 bp of 5′-UTR, an open reading frame (ORF) consisting of 1,152 and 1,179 bp of 3′-UTR. A putative polyadenylation signal (AATAAA) was recognized at position 2,435, which is located upstream of the poly (A) tail separated by 11 nucleotides. The ORF of *tyr*9 cDNA encodes a protein consisting of 383 amino acid (aa) residues with an isoelectric point of 6.80 and a predicted molecular weight of 43.72 kDa. The predicted signal peptide comprised the N-terminal sequence of 22 amino acids (Fig. 1).

### Homology and phylogenetic analyses

**Figure 2:**
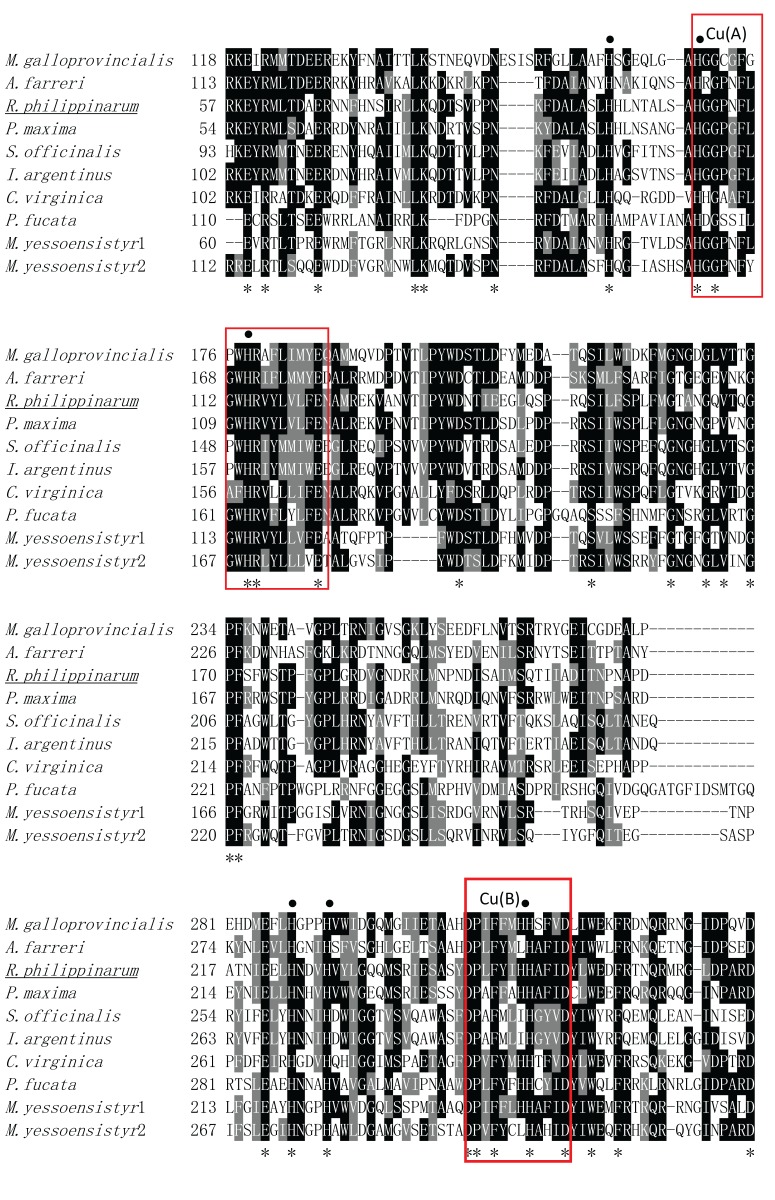
Multiple alignment of *tyr* 9 gene between the *R. philippinarum tyr* 9 and *tyr* gene of other mollusks. Identical residues were marked in dark, and similar amino acids were shaded in gray. Two copper-binding domains (CuA and CuB) were implied in red boxes. Six conserved histidine residues were labeled with ⚫. The GenBank accession numbers of the aligned sequences are: *Mytilus galloprovincialis* (OPL33388.1), *Azumapecten farreri* (ASR73340.1), *Ruditapes philippinarum* (QBC75368.1), *Pinctada maxima* (AHZ34287.1), *Sepia officinalis* (CAC82191.1), *Illex argentinus* (BAC87844.1), *Crassostrea virginica* (XP_022344539.1), *Pinctada fucata* (AAZ66340.1), *Mizuhopecten yessoensis* 1 (XP_021374237.1), and *Mizuhopecten yessoensis* 2 (XP_021373699.1).

**Figure 3:**
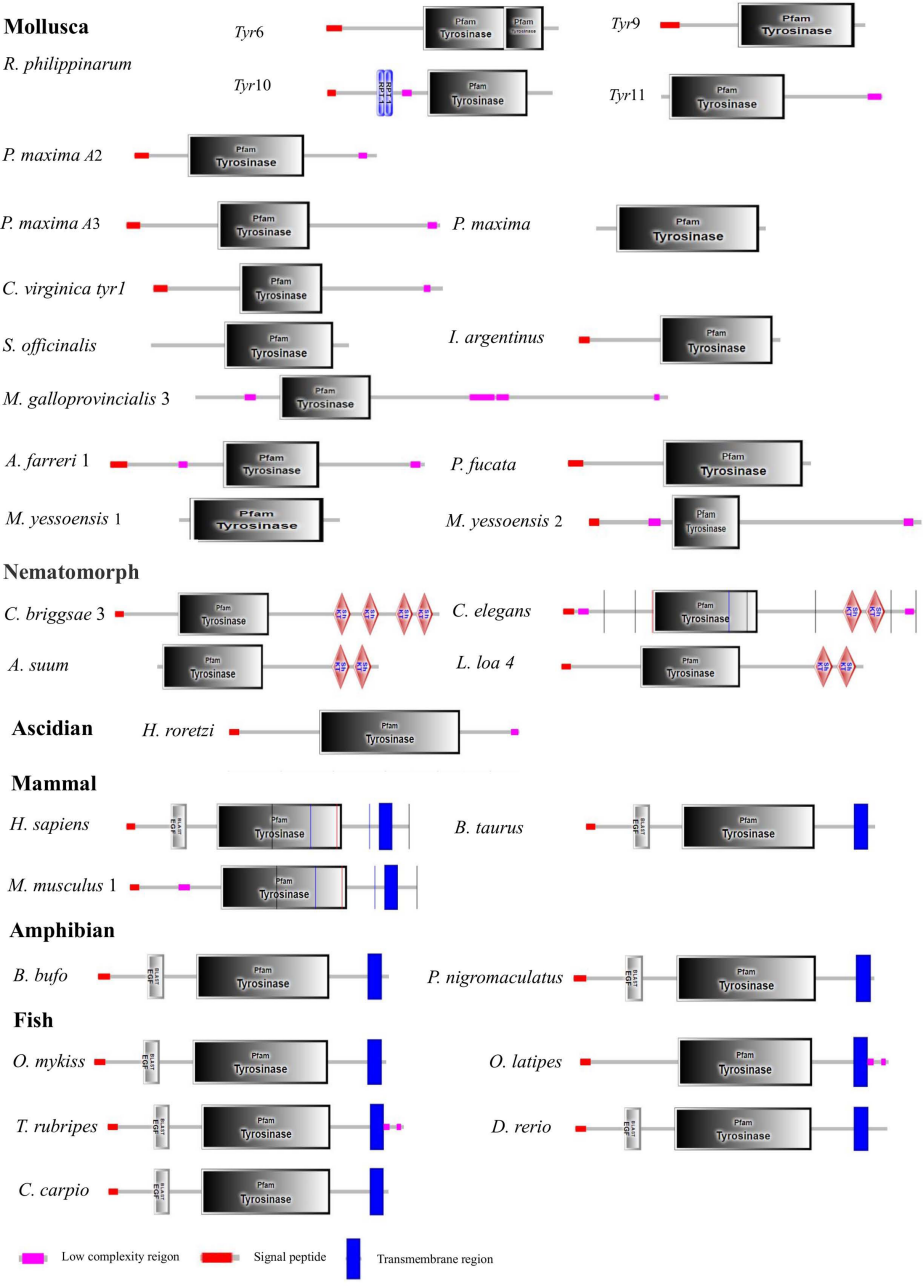
Domain architecture of tyrosinase of *R. philippinarum* and tyrosinase domain-containing of tyrosinase proteins selected from other animal. The pink block was low complexity, the red block was signal peptide, and the blue block was transmembrane region.

**Figure 4:**
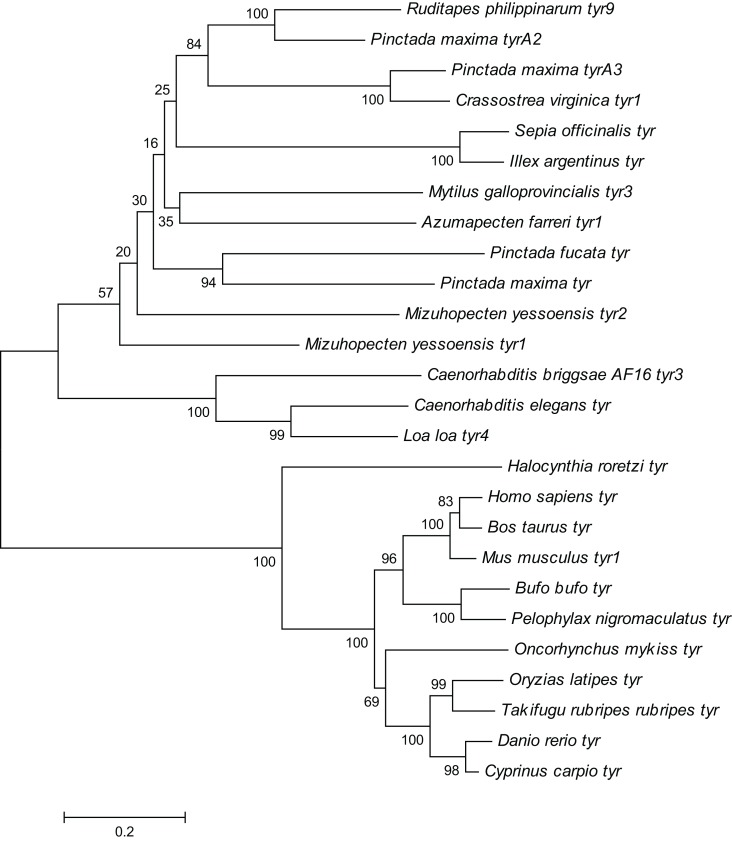
Phylogenetic tree of *R. philippinarum tyr*9 and *tyr* gene of other species was constructed with the MEGA 10.0 software using the neighbor-joining method.

The *Rptyr*9 amino acid sequence was aligned with the known amino acid sequences of their counterparts in known mollusks (Fig. 2). Two copper-binding sites Cu (A) (from His^105^ to Glu^122^) and Cu (B) (from Asp^244^ to Asp^255^) with six conserved histidine residues were found in the aligned sequences (Fig. 2). A tyrosinase domain was found in *tyr* of *R. philippinarum* and in other species (Fig. 3). To understand the evolutionary relationships between *Rptyr* and other *tyr* gene, a phylogenetic tree was constructed based on the amino acid sequences of 27 *tyr* gene (Fig. 4). The result showed that the tyrosinase domain of *Rptyr* kept a close evolutionary relationship with *tyr* gene from other mollusks, such as 60.27% with *Pinctada maxima*, 46.84% with *Crassostrea virginica*, and 42.25% with *Mytilus galloprovincialis*. As shown in Fig. 4, *R. philippinarum* clustered most closely with *Pinctada maxima*, and then with other mollusks, including *Crassostrea virginica*, *Mytilus galloprovincialis*, *Azumapecten farreri*, *Pinctada fucata*, *Mizuhopecten yessoensis*, *Sepia officinalis*, and *Illex argentinus*. The Nematomorph *tyr* gene (*Caenorhabditis briggsae*, *Caenorhabditis elegans*, *Ascaris suum*, and *Loa loa*) formed another cluster. The *tyr* gene of ascidians, mammals, fish, and amphibians formed a third cluster. Therefore, the phylogenetic relationships of the *Rptyr* amino acid sequence are consistent with traditional classification.

### *Rptyr* gene expression characteristics during larval development

**Figure 5:**
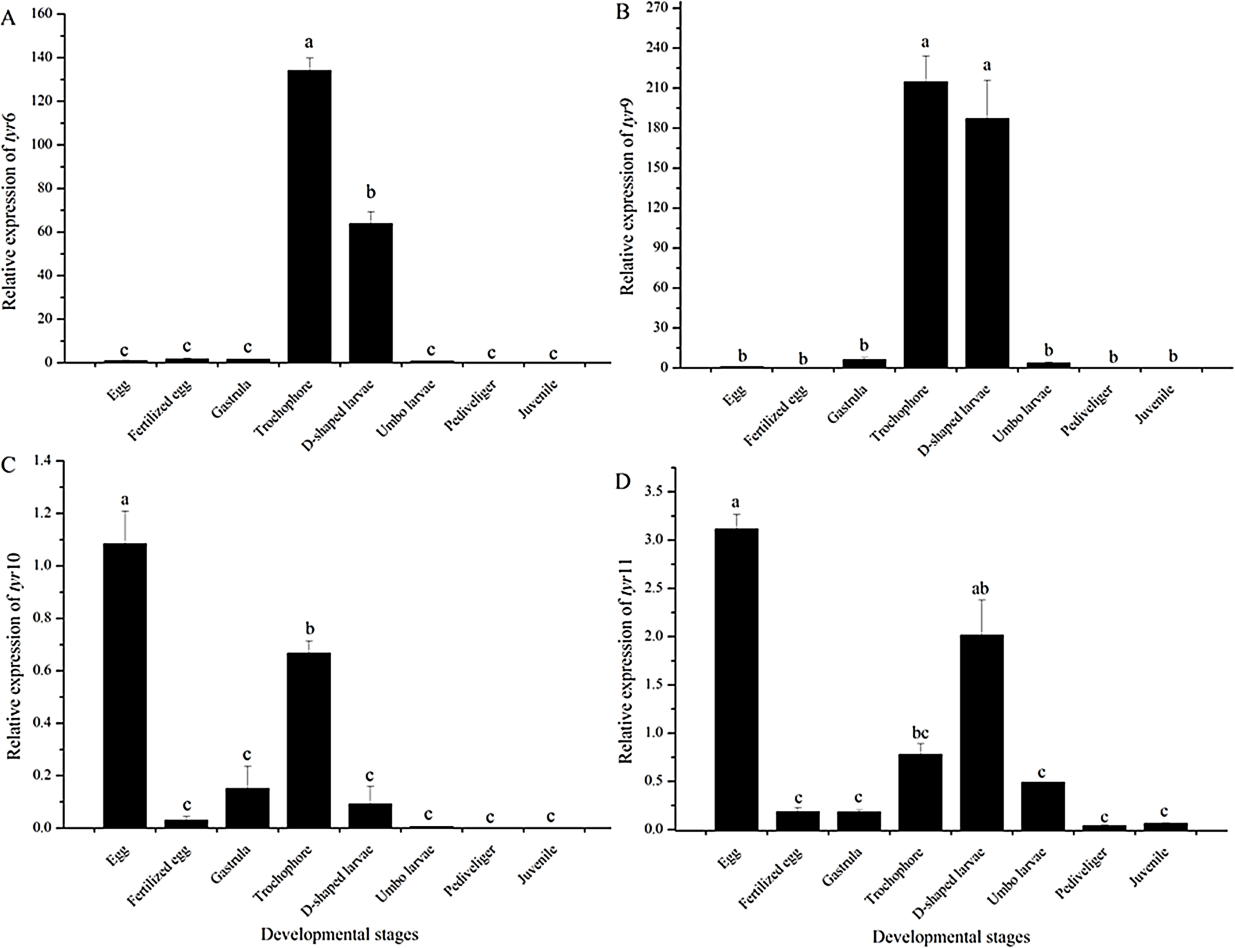
*Tyr* 6 (A), *tyr* 9 (B), *tyr* 10 (C), and *tyr* 11 (D) mRNA relative expression level in different early developmental stages (egg, fertilized egg, gastrula, trochophore, D-shaped larvae, pediveliger, and juvenile stage) of wild *R. philippinarum* detected by qRT-PCR. The β -actin gene from *R. philippinarum* was used as an internal control. Data from the qRT-PCR experiments were expressed as the mean ± SD. Bars with different letters indicate significant differences (*P* < 0.05), the same as below.

As shown in Fig. 5, the expression pattern of the *tyr* genes at different developmental stages was detected by quantitative RT-PCR analysis. The t*yr*6 and *tyr*9 genes were highly expressed at the trochophore and D-shaped stages (*P* < 0.05) (Figs. 5A and 5B). The *tyr*10 and *tyr*11 genes of *R. philippinarum* were mainly expressed at the egg, trochophore, and D-shaped larvae stage and were expressed particularly high in the egg stage (*P* < 0.05) (Figs. 5C and 5D). However, with larval development, the expression of the *tyr* gene significantly decreased and was almost undetectable in the juvenile (Fig. 5).

### Expression pattern of *Rptyr* genes in the mantle of four shell-color strains

**Figure 6:**
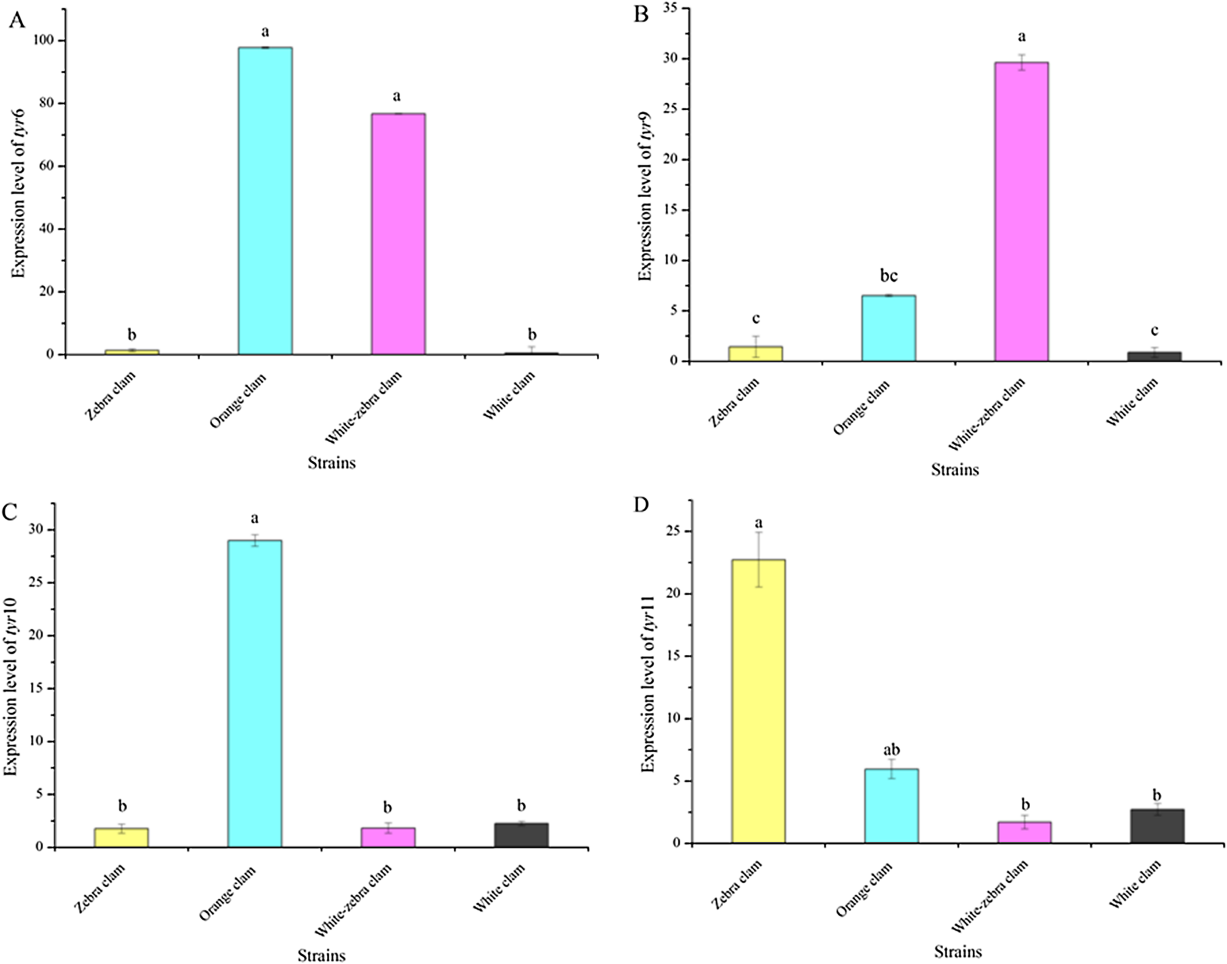
Expression analysis of *tyr*6 (A), *tyr*9 (B), *tyr*10 (C), and *tyr*11 (D) in *R. philippinarum* mantle tissues (from zebra clam, orange clam, white-zebra clam, and white clam). Expression was determined with qRT-PCR relative to β*-actin* mRNA expression. Bars with different letters indicate significant differences (*P* < 0.05).

To investigate the relationship between the expression level of *tyr* genes and different shell colors, the expression level of *tyr* genes in the adults of four shell-color strains were analyzed. *Tyr*6, *tyr*9, *tyr*10, and *tyr*11 genes were expressed in the mantle of different strains with various patterns (Figs. 6A−6D). Zebra clams had a high *tyr*11 gene expression level, which was significantly different from white-zebra clams and white clams (*P* < 0.05). Orange clams had a high expression level in the mantle of the *tyr*6, *tyr*9, *tyr*10, and *tyr*11 gene (*P* < 0.05). White-zebra clams had significantly high expression levels of *tyr*6 and *tyr*9 (*P* < 0.05). Only the white clam expressed a low level of the four *tyr* gene (*P* < 0.05). According to these results, the *tyr* gene were more highly expressed in the mantle of dark shell-color strains (orange, zebra, and white-zebra clams) than in the light shell-color strain (white clams).

### The tissue distribution of *Rptyr* transcripts

**Figure 7:**
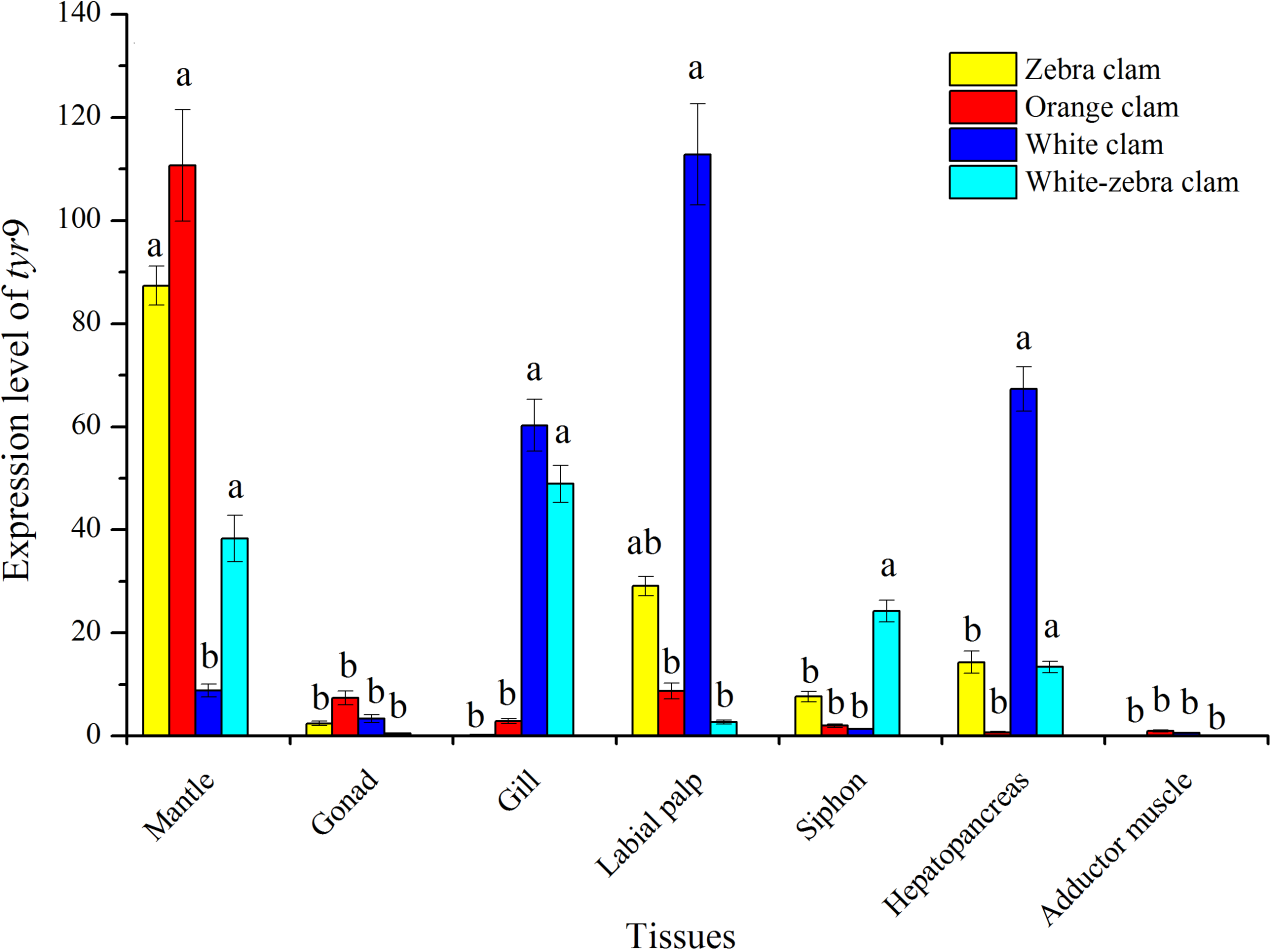
Expression analysis of *tyr*9 mRNA in the mantle, gonad, gill, labial palp, siphon, hepato pancreas, adductor muscle of *R. philippinarum* detected with qRT-PCR. Bars with different letters indicate significant differences (*P* < 0.05).

The tissue-specific expression of *tyr* genes in different shell-color strains of *R. philippinarum* was analyzed using qRT-PCR. In this study, the transcript of *tyr*9 was expressed in a wide range of the tissues examined, including mantle, gonad, gill, labial palp, siphon, hepatopancreas, and adductor muscle (Fig. 7). In zebra clams, *tyr*9 was mainly expressed in the mantle and labial palp (*P* < 0.05). In orange clams, *tyr*9 was only detected in the mantle at a significantly high level (*P* < 0.05). In white-zebra clams, *tyr*9 was mainly expressed in the mantle, gill, and hepatopancreas (*P* < 0.05). White clams had a high expression level in the gill, labial palp, and hepatopancreas (*P* < 0.05).

### RNAi-mediated *tyr*9 knockdown in *R. philippinarum*

**Figure 8:**
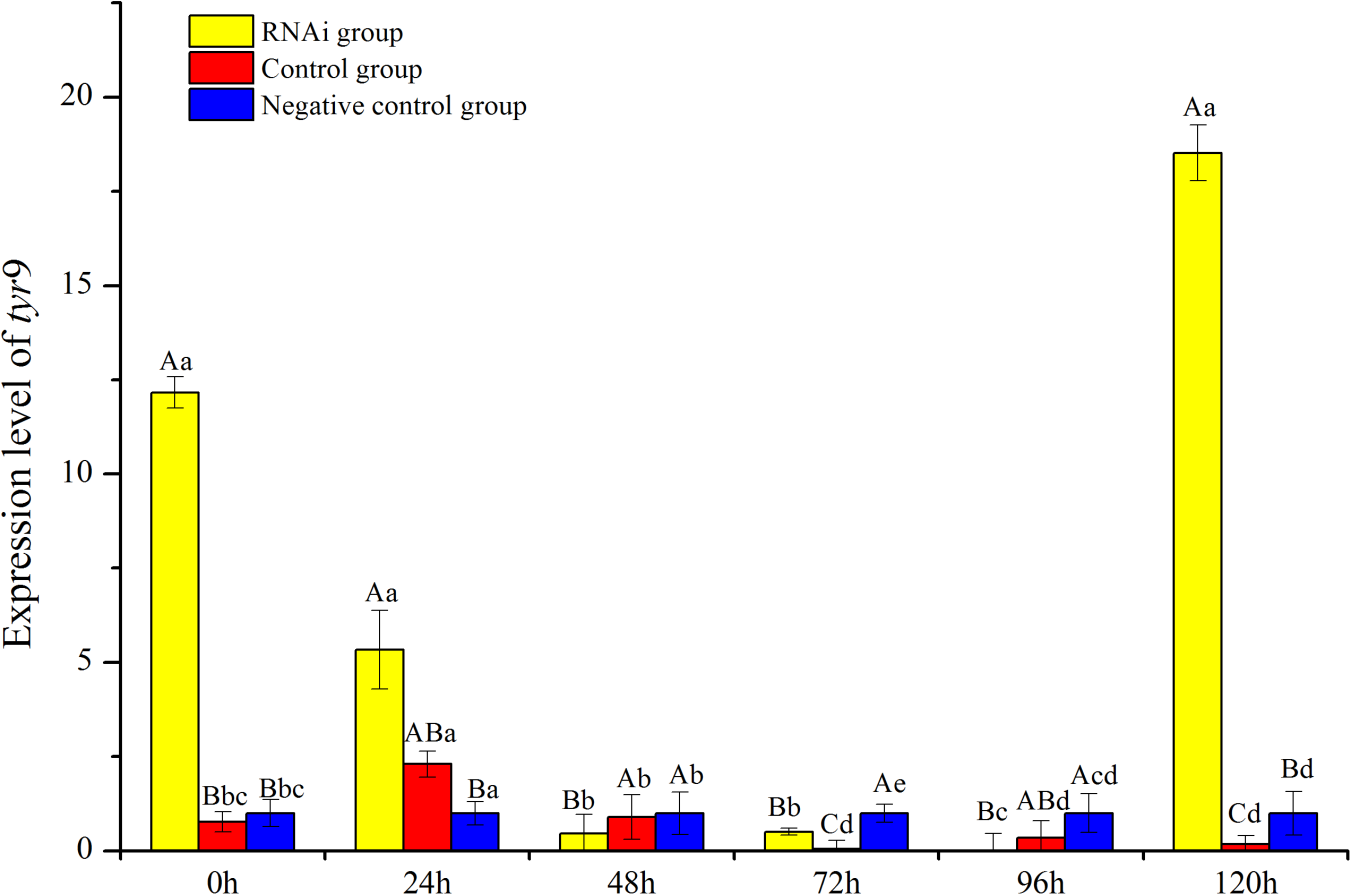
Analysis of expression difference of different times under RNA interference of *R. philippinarum*. The capital letters indicated significant differences at the same time points with different processing, the lowercase letters meant significant differences at different time point with same processing.

In this study, the temporal expression of *Rptyr*9 in healthy clams after dsRNA injection was detected. The transcriptional characteristics of *Rptyr*9 in the mantle after dsRNA injection are shown in Fig. 8. The expression level of *Rptyr*9 mRNA was significantly downregulated in the mantle by 2.28 and 23.94-fold at 24 and 72 h (*P* < 0.05), respectively, and was hardly detected at 96 h (*P* < 0.05) after RNAi. However, the transcripts of *tyr*9 recovered to its original level (1.52-fold higher than the original level) at 120 h (Fig. 8). While both the control and negative control groups showed a stable expression level of *tyr*9 similar to the original level during the whole experiment.

## Discussion

Currently, tyrosinases are considered to be involved in many biological activities of mollusks, including non-calcified shell formation (Yang et al., 2017; Huan et al., 2013), shell growth (Feng, Li & Yu, 2019), pigmentation (Feng et al., 2015; Chen et al., 2016; Jing, 2015; Yu et al., 2018), and the immune response (Zhou et al., 2012; Asokan, Arumugam & Mullainadhan, 1997). Studies of the *tyr* gene expression pattern in *Crassostrea gigas* (Yang et al., 2017) and *Crassostrea angulata* (Huan et al., 2013) at the early developmental stages found that *tyr* was highly expressed at the trochophore stage until the D-veliger stage, which indicates that the *tyr* gene might participate in the formation of initial non-calcified shell. In addition, several studies have suggested that tyrosinase plays a key role in melanin synthesis and the color formation of the *Pteria penguin* (Yu et al., 2018). Studies on *Crassostrea gigas* (Feng et al., 2015) and *H. cumingii* (Chen et al., 2016) have shown that the dark shell-color variants (golden and purple, respectively) represented a higher expression level of tyrosinase than other shell-color variants, which indicates that tyrosinase might play an important role in the pigmentation of shellfish.

In this study, we detected the expression level of the *tyr*9 gene in different tissues of four shell-color strains. Our data showed that the *tyr*9 gene was mainly expressed in the mantle of dark shell-color strains (orange, zebra, and white-zebra clams) compared with white clams. Among them, the orange clams had the highest expression level of *tyr*9, and then the zebra and white-zebra clams. However, in the white clam, a light shell-color strain, *tyr*9 was mainly expressed in the gill, labial palp, and hepatopancreas. Moreover in the mantle, *tyr*6, *tyr*9, *tyr*10, and *tyr*11 mainly expressed in the dark shell-color strains compared with light shell-color clams. Similar results were found in *H. cumingii*, *HcTyr* was more highly expressed in the nacre of the purple strain than in the white strain (Chen et al., 2016). Therefore, our study indicated that *tyr* gene expression was associated with the accumulation of melanins to form various shell colors and the expression of *tyr* gene in the mantle increased with the darkness of the shell color.

In the past decade, a number of studies mainly focused on the roles of tyrosinases in adult shell calcification and pigmentation in Mollusca (Meinhardt & Klingler, 1987; Wada & Komaru, 1996; Comfort, 2010), but the expression level of tyrosinases in early developmental stages is largely unexplored. In the present study, we found *tyr*10 and *tyr*11 showed a high expression level in the egg stage which could be inferred to be from maternal contribution (Wang et al., 2015). *Tyr6*, *tyr*9, *tyr*10, and *tyr*11 were highly expressed at the trochophore and D-shaped larvae stages and greatly down-regulated after the D-shaped larvae stage, which is similar with the expression profiles of tyrosinase genes in early larva of *Crassostrea angulata* and *Crassostrea gigas* (Yang et al., 2017; Huan et al., 2013). These results suggested that *tyr*6, *tyr*9, *tyr*10, and *tyr*11 might play important roles in the formation of the primary larval shells of Manila clams.

RNA interference is an important molecular tool for the analysis of gene function in vivo (Feng, Li & Yu, 2019). Over the past decades, RNAi was widely used to specifically silence the expression of any gene to study its functional effect (Wheeler, Carpenter & Sabatini, 2005) and as an effective approach for gene function validation and analysis (Paschka et al., 2003). In this work, RNAi-mediated gene silencing technology was performed to validate the effects of RNAi and the gene function. The results showed that the *Rptyr*9 gene was silenced at 48 to 96 h post-injection and increased gradually at 96 h post-injection and recovered to its original level at 120 h, which indicates that the endogenous *tyr*9 mRNA was degraded by dsRNA. However, there was no obvious change in the shell-color phenotypes of the clams. These results suggested that the dsRNA of *tyr*9 could effectively reduce the expression level of *tyr*9 at 48 to 96 h post-injection, but shell-color determination could be the long-term result of melanin accumulation (Yu et al., 2018), short-term knockdown of the *tyr* gene may not rapidly change the phenotype. A recent study in *Crassostrea gigas* reported the expression of tyrosinase was knocked down and shell growth was hindered after the dsRNA of tyrosinase was fed for 35 days (Feng, Li & Yu, 2019).

## Conclusion

We identified four tyrosinase genes (*tyr*6, *tyr*9, *tyr*10, and *tyr*11) from *R. philippinarum*. Tissue expression analysis showed that *tyr* genes were highly expressed in mantle, a shell formation and pigmentation-related tissue. Those four *tyr* genes were expressed highly mainly in the mantle of dark shell-color strains and the expression level increased with the darkness of the shell-color. The injection of dsRNA of *tyr*9 significantly inhibited *tyr*9 expression temporarily. Therefore, we believe that tyrosinases play key roles in shell formation and high tyrosinase gene expression contributes to melanin accumulation to form a dark shell-color in *R. philippinarum*.

## Supplemental Information

10.7717/peerj.8641/supp-1 Supplemental Information 1 Four shell-color strains of Manila clam.

10.7717/peerj.8641/supp-2 Supplemental Information 2 Try qPCR experiment raw data.

## Additional Information and Declarations

### DNA Deposition

The following information was supplied regarding the deposition of DNA sequences: The cDNA sequence of *tyr*9 gene (designated as *Rptyr*9) of *R. philippinarum* was obtained using the RACE method and is available in GenBank: MH392190.

### Data Availability

The following information was supplied regarding data availability: The raw measurements are available in the Supplemental Files.
