# Supplementary figures and images for: Molecular cloning and expression analysis of tyrosinases (tyr) in four shell-color strains of Manila clam Ruditapes philippinarum
Source: PeerJ. 2020 Feb 17;8:e8641. doi: 10.7717/peerj.8641 (PMC7032058; doi:10.7717/peerj.8641)

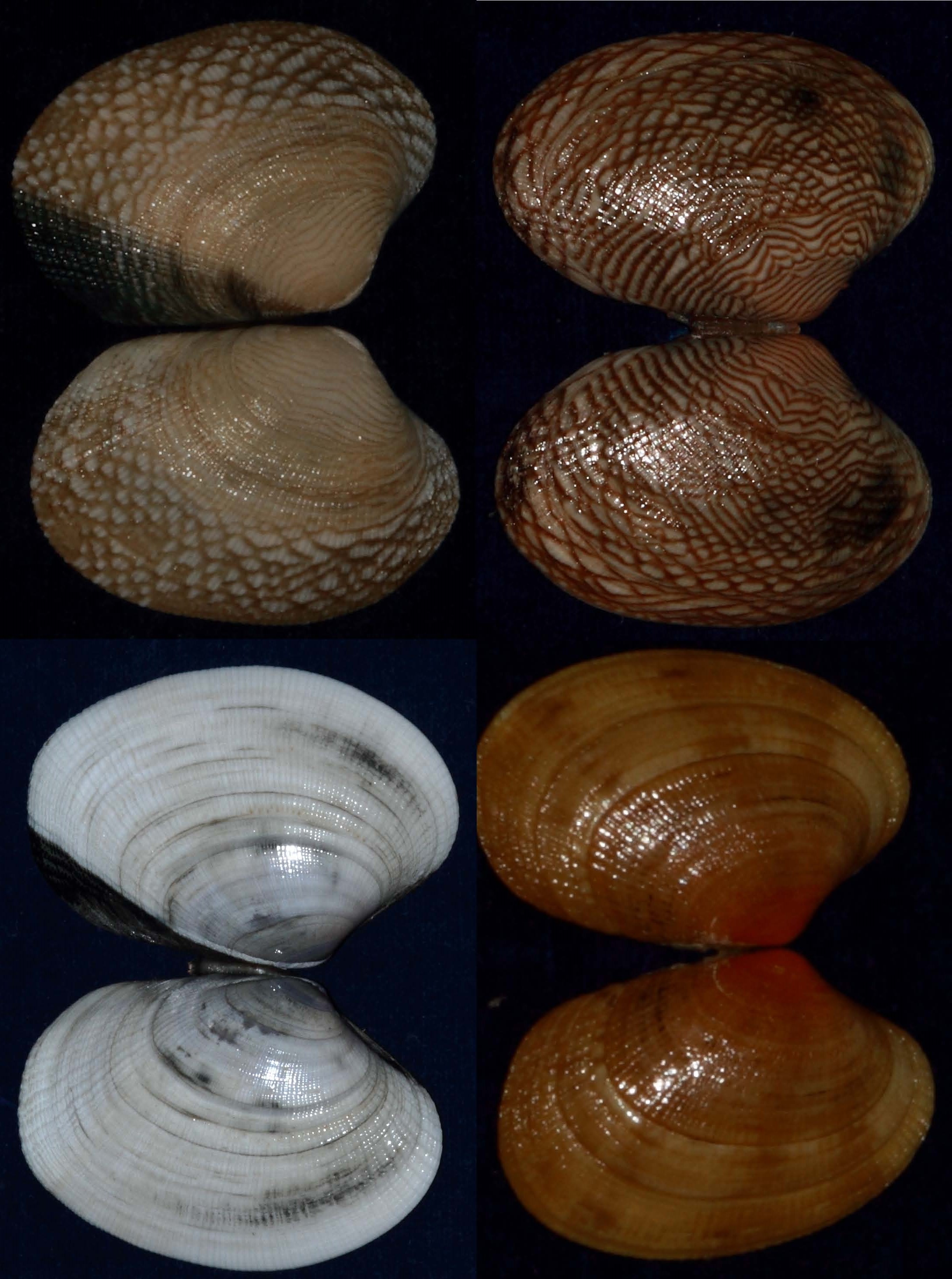

Supplement: Supplemental Information 1 [file peerj-08-8641-s001.jpg]
